# Ambulatory circadian monitoring in sleep disordered breathing patients and CPAP treatment

**DOI:** 10.1038/s41598-021-94315-0

**Published:** 2021-07-19

**Authors:** Antonio Martinez-Nicolas, Marc Guaita, Joan Santamaría, Josep M. Montserrat, Juan Antonio Madrid, María Angeles Rol

**Affiliations:** 1 Chronobiology Lab, Department of Physiology, College of Biology, University of Murcia, Mare Nostrum Campus. IUIE. IMIB - Arrixaca, 30100 Espinardo, Murcia Spain; 2 Ciber Fragilidad y Envejecimiento Saludable (CIBERFES), Madrid, Spain; 3 Multidisciplinary Sleep Disorders Unit, Hospital Clinic of Barcelona, Barcelona, Spain; 4 Neurology Department, Hospital Clinic of Barcelona, Barcelona, Spain; 5 Pneumology Department, Hospital Clinic of Barcelona, Barcelona, Spain; 6 Institut d’Investigacions Biomèdiques August Pi i Sunyer (IDIBAPS), Barcelona, Spain; 7 Ciber Enfermedades Respiratorias (CIBERES), Madrid, Spain

**Keywords:** Physiology, Circadian rhythms and sleep

## Abstract

Our aim was to evaluate the circadian rhythm of motor activity, body position and integrated variable TAP (composed by wrist **T**emperature, motor **A**ctivity and body **P**osition) in Sleep Disordered Breathing (SDB), its relation to SDB severity and the effect of continuous positive airway pressure (CPAP) on these circadian rhythms. To do this, we monitored motor activity and body position rhythms of 78 SDB patients (53.3 ± 1.2 years old, 26.9% women) and 32 healthy subjects (51.4 ± 3.2 years old, 43.8% women) for 1 week. On the last day of that week, SDB patients underwent a polysomnography followed by a Maintenance of Wakefulness Test, Multiple Sleep Latency Test and Sustained Attention to Response Task protocol. A subgroup of 18 moderate to severe SDB patients was treated with CPAP and monitored again after 3 months under treatment. A non-parametrical analysis was performed to characterize the circadian patterns to assess differences between groups and associations between sleep and circadian parameters. Circadian variables were altered in SDB, exhibiting a direct relationship to SDB severity. The motor activity pattern showed a clear improvement with CPAP treatment. Thus, circadian ambulatory monitoring, including the integrated variable TAP, could be used to evaluate the circadian alterations caused by SDB and activity pattern to monitor the effect of CPAP treatment.

## Introduction

The circadian system is the main responsible for the temporal regulation of most physiological processes, including thermoregulation, metabolism or the sleep–wake cycle^1^. Among these processes, the sleep–wake cycle is regulated by an homeostatic sleepiness accumulation, a circadian process and sleep inertia^2, 3^.

The increasing prevalence of SDB through the world has become a major health concern, due to its multiple consequences including hypertension, diabetes, cardiovascular and cerebrovascular disease, cognitive impairment and even cancer^4^. The chronodisruptive effect of SDB is supported by both the circadian system alteration at several levels, such as thermoregulation or the sleep–wake cycle itself^5–8^, and the circadian restoration of sleep, blood pressure, thermoregulation, immune response or haemostatic system by Continuous Positive Airway Pressure (CPAP)^5, 6, 9–13^. Also, this relationship seems bidirectional since the circadian system modulates the incidence and length of apnoea events^14^.

The rest-activity marker rhythm is inherently associated to the sleep–wake cycle^15^, used as choice method for circadian sleep disorders assessment^16^ and has been validated against polysomnography in SDB patients^17^. However, isolated marker rhythms are submitted to masking, thus multivariable recordings^15, 18, 19^ and variable integration have been developed^15^. In this sense, the integrated variable TAP (composed by wrist **T**emperature, motor **A**ctivity and body **P**osition) developed for sleep detection and successfully validated against polysomnography^15, 20^, can improve the accuracy provided by a single marker rhythm^15, 21^.

Thus, the main purpose of this study was to evaluate the circadian rhythm of motor activity, body position and the integrated TAP variable in order to determine how these rhythms are affected by sleep disordered breathing severity and continuous positive airway pressure treatment, and in consequence Ambulatory Circadian Monitoring (ACM) could constitute a screening tool for this disorder as well as CPAP efficacy under free-living conditions.

## Results

### Clinical and polysomnographic characteristics

From the consecutive SDB patients originally studied (n = 98), twenty were excluded due to the following reasons: irregular sleep–wake rhythms (n: 3), acute sleep deprivation prior to the sleep study (n: 1), REM sleep without atonia (n: 1), severe depressive symptoms (n: 1), migraines during the nap protocol (n: 1) and removing the sensors (i.e. insufficient data for analysis) either at home or during the PSG procedure (n: 13). Then, the group finally comprised 78 adults with a wide spectrum of disease (see our previous work^5^ for clinical and polysomnographic characteristics). With regard to the 30 patients requiring CPAP, one refused to complete the protocol and 11 presented non valid recordings (removing the sensors or insufficient monitoring time); therefore, 18 moderate-to-severe SDB patients receiving CPAP were revaluated at least 6 weeks after the baseline study. PSG revealed a complete resolution of SDB in all patients and improved excessive daytime sleepiness measured by ESS, BSI sleepiness index, MSLT-SL, MWT-SL and MWT-E. However, CPAP treatment did not improve SART errors (see Martinez-Nicolas et al.^5^).

### Circadian characteristics of sleep disordered breathing

**Figure 1:**
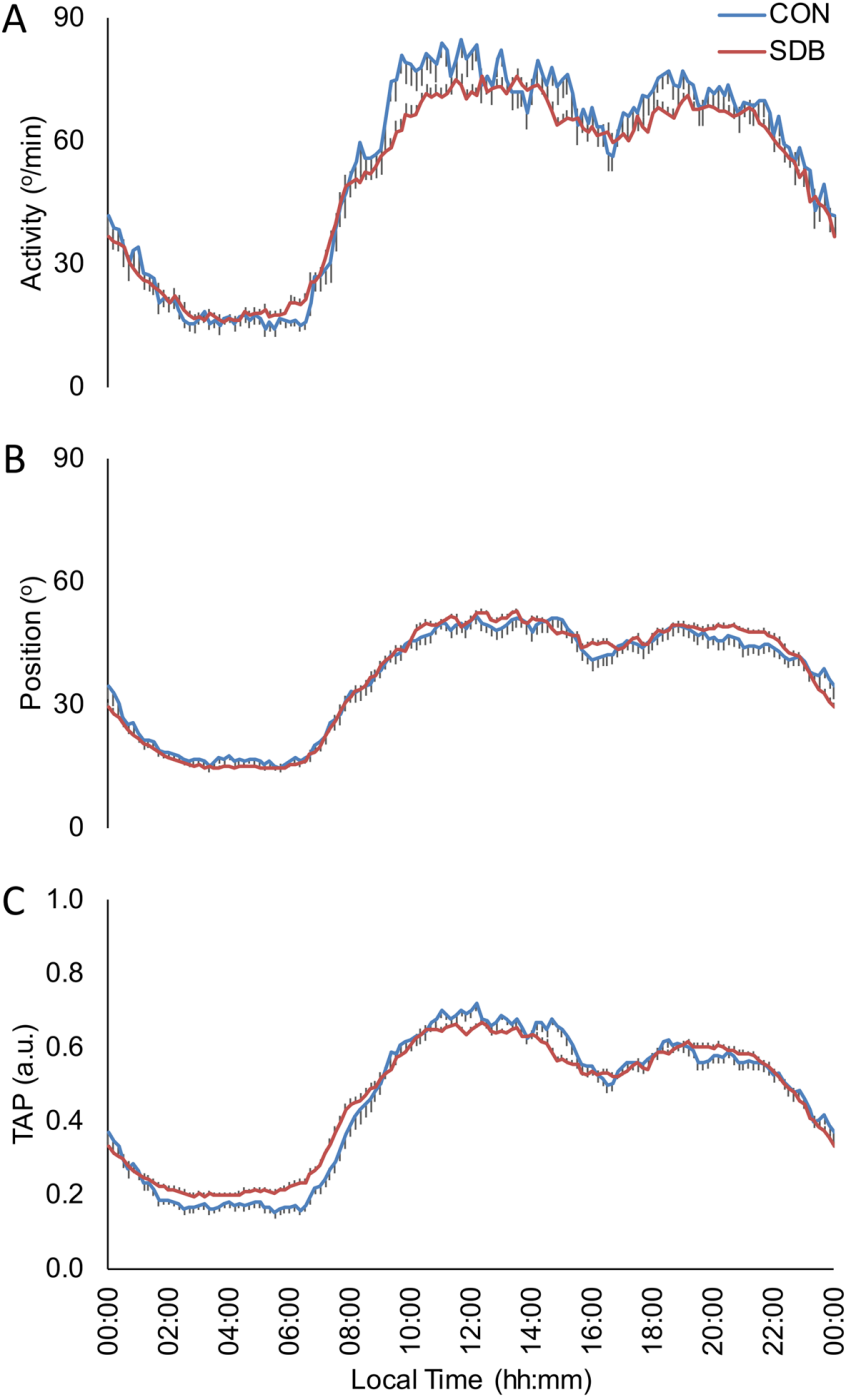
Mean waveforms for SDB patients (red line, n: 78) and control subjects (blue line, n: 32) for: (**A**) Activity (º/min), (**B**) Body Position (º) and (**C**) integrated variable TAP (a. u.). All data are expressed as mean ± SEM.

Daily mean patterns and circadian parameters of motor activity, body position and TAP variable for SDB patients (n: 78) and healthy subjects (n: 32) are shown in Fig. 1 and Table 1. When comparing rhythms for motor activity of SDB patients and healthy subjects, SDB patients showed lower circadian stability as indicated by decreased IS (General Linear Model controlled by age, sex and BMI; p < 0.05), as well as higher RA and daytime values (M10) and a phase advance in TL5 for body position than healthy subjects. Finally, TAP pattern (and index of general activation) of SDB patients showed lower IS, CFI and daytime values (M10) compared with healthy subjects (General Linear Model controlled by age, sex and BMI; p < 0.05).

**Table 1:** Non-parametrical analysis of the circadian rhythms for activity, position and TAP in healthy subjects (n: 32) and SDB patients (n: 78).

Activity		IS	IV	RA	CFI	TL5 (hh:mm)	TM10 (hh:mm)	L5 (^o^/min)	M10 (^o^/min)
	Control	0.51 ± 0.02	0.63 ± 0.02	0.67 ± 0.02	0.62 ± 0.01	03:57 ± 00:14	15:34 ± 00:27	14.86 ± 0.92	76.74 ± 2.16
	SDB	0.41 ± 0.01	0.73 ± 0.02	0.62 ± 0.01	0.55 ± 0.01	04:04 ± 00:06	15:12 ± 00:13	16.84 ± 0.76	70.50 ± 1.40
	*p*	*0.000*	*0.117*	*0.765*	*0.084*	*0.582*	*0.150*	*0.795*	*0.541*
Position		IS	IV	RA	CFI	TL5 (hh:mm)	TM10 (hh:mm)	L5 (^o^)	M10 (^o^)
	Control	0.58 ± 0.03	0.30 ± 0.02	0.53 ± 0.03	0.65 ± 0.02	04:53 ± 00:37	15:06 ± 00:25	14.90 ± 0.83	48.93 ± 1.70
	SDB	0.55 ± 0.01	0.31 ± 0.01	0.55 ± 0.01	0.65 ± 0.01	04:16 ± 00:06	15:27 ± 00:11	14.59 ± 0.47	49.53 ± 0.54
	*p*	*0.237*	*0.865*	*0.021*	*0.103*	*0.013*	*0.191*	*0.204*	*0.026*
TAP		IS	IV	RA	CFI	TL5 (hh:mm)	TM10 (hh:mm)	L5 (a. u.)	M10 (a. u.)
	Control	0.73 ± 0.01	0.26 ± 0.02	0.60 ± 0.02	0.74 ± 0.01	03:54 ± 00:12	14:48 ± 00:15	0.16 ± 0.01	0.64 ± 0.01
	SDB	0.56 ± 0.01	0.31 ± 0.01	0.52 ± 0.01	0.64 ± 0.01	03:46 ± 00:07	14:48 ± 00:04	0.20 ± 0.01	0.61 ± 0.00
	*p*	*0.000*	*0.845*	*0.590*	*0.018*	*0.599*	*0.866*	*0.514*	*0.017*

Interdaily stability (IS); intradaily variability (IV); relative amplitude (RA); circadian function index (CFI); mean of the 10 consecutive hours with the highest values (M10) and its timing (TM10); mean of the 5 consecutive hours with the lowest values (L5) and its timing (TL5). Values are expressed as the mean ± SEM. Significant differences between healthy and SDB subjects are highlighted in bold (p < 0.05, General Linear Model controlled for gender, age and BMI).

**Figure 2:**
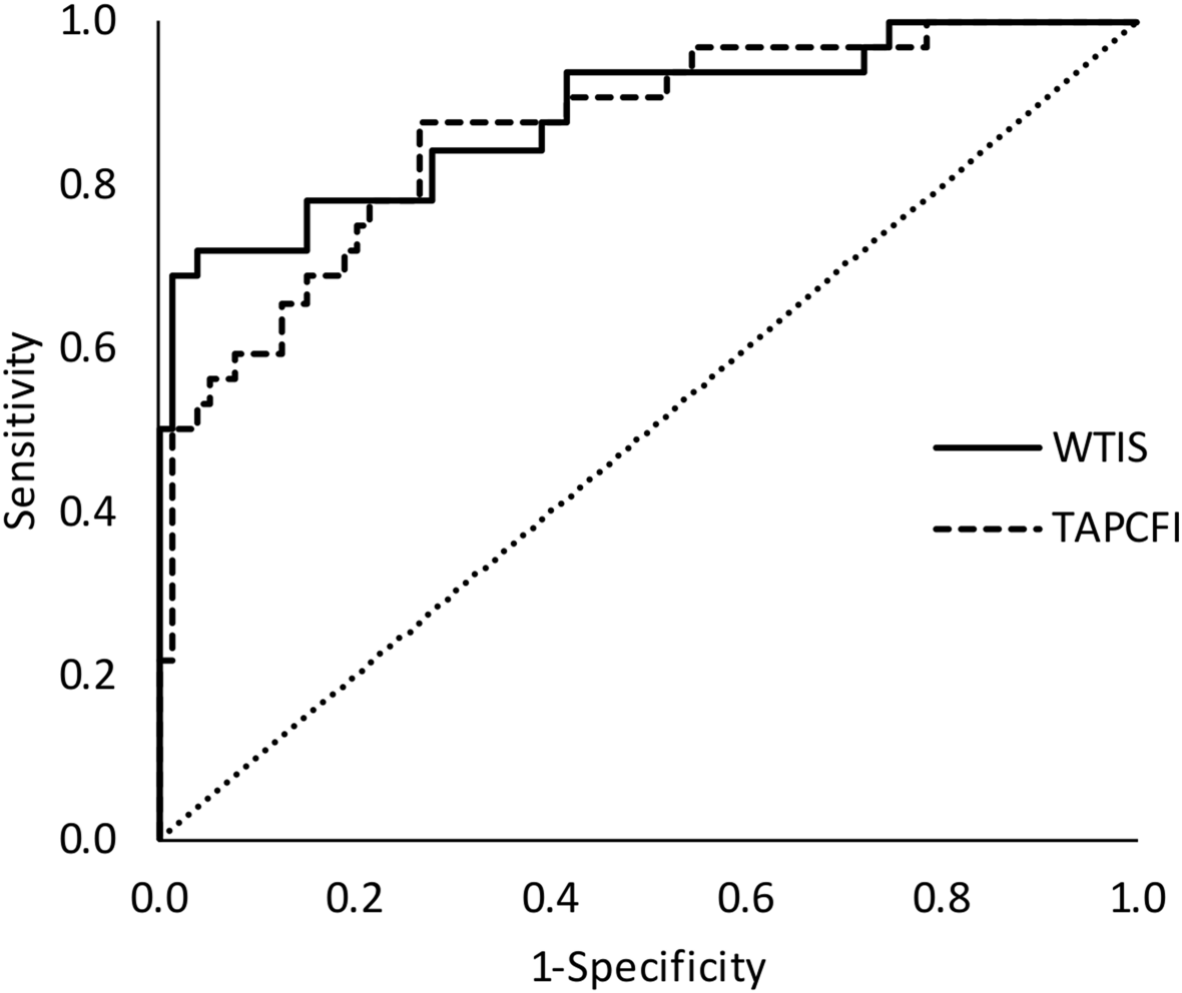
ROC Curves for Diagnostic ability to detect SDB patients (n: 78) against controls (n: 32) according to wrist temperature stability (WTIS, continuous line) versus TAP robustness (TAPCFI, discontinuous line).

The decision tree to discern between healthy subjects (n: 32) and SDB patients (n: 78) selected WT stability (IS) with a maximum agreement rate (89.2%) at 0.53 (sensitivity = 88.5%; specificity = 91.7%; positive predictive value = 97.5%; negative predictive value = 68.8%). The ROC curve shows an area under the curve of 0.89 (p < 0.001) as shown in Fig. 2. The second most discriminant variable chosen by the decision tree was the TAP robustness (CFI) with a cut-off point of 0.79 (Fig. 2), reached an agreement rate of 80.2% (sensitivity = 79.4%; specificity = 85.7%; a positive predictive value = 97.5%; negative predictive value = 37.5%) and the ROC area under the curve was 0.794 (p < 0.001).

### Circadian impairment by sleep disordered breathing severity

**Figure 3:**
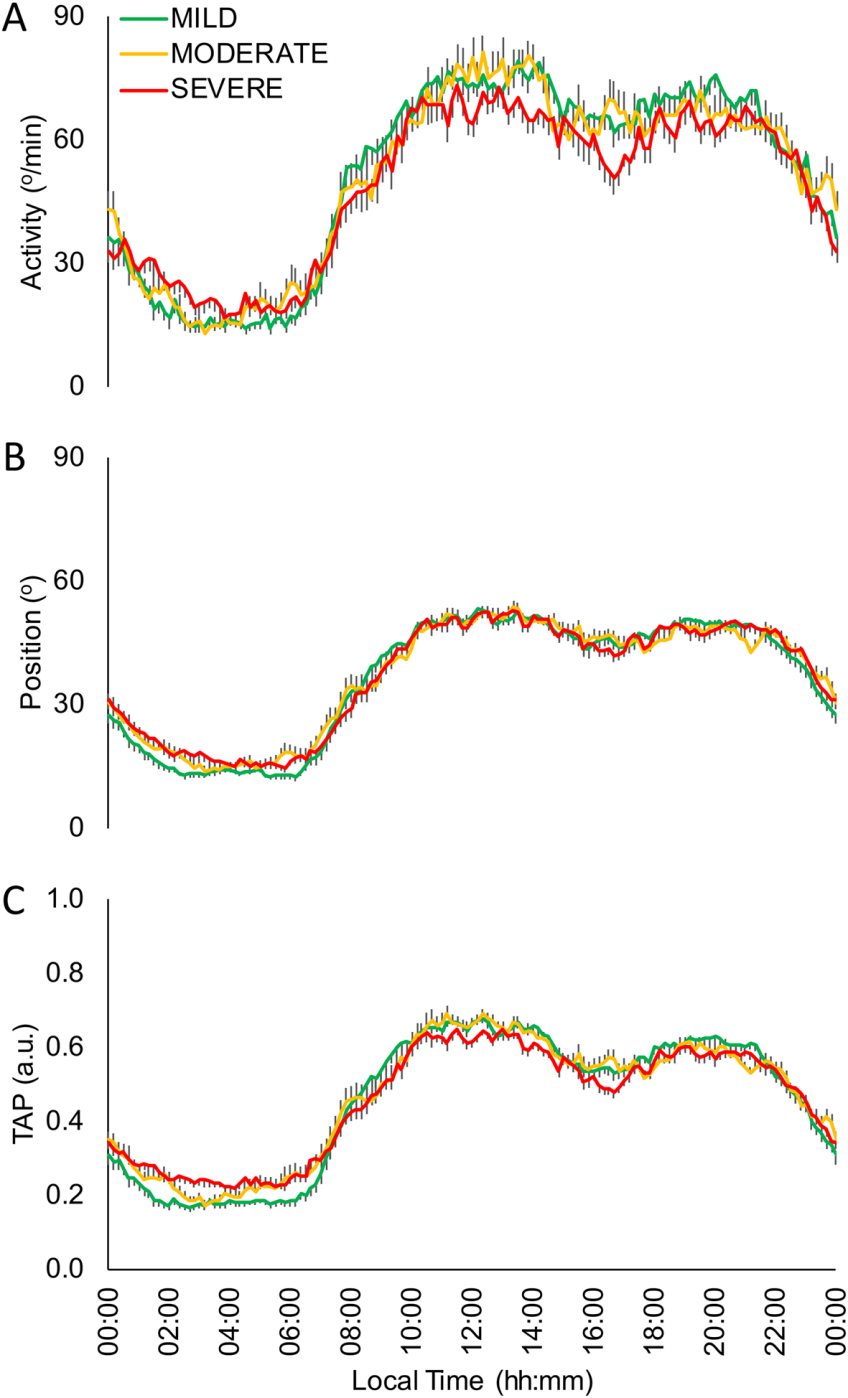
Mean waveforms for mild, moderate and severe SDB subjects according to their Apnoea/Hypopnoea Index for: (**A**) Activity (º/min), (**B**) Body Position (º) and (**C**) integrated variable TAP (a. u.). Snorer**/**Mild group: subjects with less than 15 events/hour (green line, n: 31); Moderate: between 15 and 30 events/hour (yellow line, n: 19); Severe: more than 30 events/hour (red line, n: 28). All data are expressed as mean ± SEM.

The SDB severity effect on the circadian rhythmic parameters (Fig. 3) was addressed by comparing snorers/mild (n: 31), moderate (n: 19) and severe SDB (n: 28) by a General Linear Model controlled for age, sex and BMI, and followed by a Bonferroni’s post hoc analysis (Table 2). The motor activity pattern of snorers/mild apnoea group compared to severe group (Fig. 3A and Table 2) showed a more stable pattern and lower values at night (L5), this latter also occurred for body position rhythm together with a phase advance (TL5) and higher day/night contrast (RA) (Fig. 3B and Table 2), presenting moderate group intermediate values. Finally, the integrated variable TAP showed a more stable (IS) and robust pattern (CFI) in the snorers/mild apnoea group compared to severe group (Fig. 3C and Table 2), being the moderate group in an intermediate status. In addition, the mild/snorers group showed lower N1, AI, AHI, CT90, ODI3, BSI and higher N3, REM, REM Episodes, MSAT, NSAT and MWT-E than severe SDB group, with moderate group again with intermediate values as it was previously published^5^.

**Table 2:** Non-parametrical analysis of the circadian patterns for activity, position and TAP according to SDB severity.

Activity		IS	IV	RA	CFI	TL5 (hh:mm)	TM10 (hh:mm)	L5 (^o^/min)	M10 (^o^/min)
	Mild	0.45 ± 0.01	0.69 ± 0.02	0.65 ± 0.02	0.58 ± 0.01	03:55 ± 00:09	15:15 ± 00:24	14.18 ± 1.08	70.22 ± 2.28
	Moderate	0.41 ± 0.02	0.72 ± 0.03	0.61 ± 0.02	0.55 ± 0.02	04:04 ± 00:13	15:04 ± 00:24	17.63 ± 1.40	72.34 ± 2.68
	Severe	0.37 ± 0.02*	0.77 ± 0.03	0.59 ± 0.02	0.53 ± 0.02	04:15 ± 00:11	15:12 ± 00:20	19.30 ± 1.14*	69.56 ± 2.44
	*p*	*0.003*	*0.080*	*0.163*	*0.187*	*0.386*	*0.977*	*0.006*	*0.723*
Position		IS	IV	RA	CFI	TL5 (hh:mm)	TM10 (hh:mm)	L5 (^o^)	M10 (^o^)
	Mild	0.57 ± 0.02	0.29 ± 0.01	0.58 ± 0.02	0.67 ± 0.01	04:04 ± 00:09	15:38 ± 00:19	12.70 ± 0.68	48.41 ± 0.85
	Moderate	0.53 ± 0.03	0.34 ± 0.02	0.51 ± 0.02*	0.62 ± 0.02	03:59 ± 00:14	15:14 ± 00:25	15.83 ± 0.88*	49.30 ± 1.01
	Severe	0.56 ± 0.02	0.31 ± 0.02	0.53 ± 0.02	0.64 ± 0.02	04:42 ± 00:09*	15:23 ± 00:17	15.92 ± 0.72*	50.98 ± 0.91
	*p*	*0.415*	*0. 301*	*0.041*	*0.078*	*0.006*	*0.946*	*0.002*	*0.162*
TAP		IS	IV	RA	CFI	TL5 (hh:mm)	TM10 (hh:mm)	L5 (a. u.)	M10 (a. u.)
	Mild	0.60 ± 0.02	0.28 ± 0.02	0.55 ± 0.02	0.67 ± 0.01	03:37 ± 00:12	14:44 ± 00:17	0.18 ± 0.01	0.62 ± 0.01
	Moderate	0.54 ± 0.02*	0.31 ± 0.02	0.51 ± 0.02	0.63 ± 0.02	03:39 ± 00:13	14:38 ± 00:20	0.20 ± 0.01	0.62 ± 0.01
	Severe	0.53 ± 0.02*	0.34 ± 0.02	0.48 ± 0.02	0.62 ± 0.01*	04:00 ± 00:13	14:58 ± 00:19	0.22 ± 0.01	0.61 ± 0.01
	*p*	*0.014*	*0.169*	*0.069*	*0.026*	*0.363*	*0.725*	*0.057*	*0.590*

Snorer/Mild: subjects with AHI lower than 15 (n: 31); Moderate: AHI higher than 15 and lower than 30 (n: 19); Severe: AHI higher than 30 (n: 28). Interdaily stability (IS); intradaily variability (IV); relative amplitude (RA); circadian function index (CFI); mean of the 10 consecutive hours with the highest values (M10) and its timing (TM10); mean of the 5 consecutive hours with the lowest values (L5) and its timing (TL5). Values are expressed as the mean ± SEM. p denotes global significance level according to General Linear Model (in bold probability values lower than 0.05). * Indicates statistical differences when compared to mild SDB patients (General Linear Model controlled for gender, age and BMI followed by a Bonferroni’s post hoc, p < 0.05).

**Figure 4:**
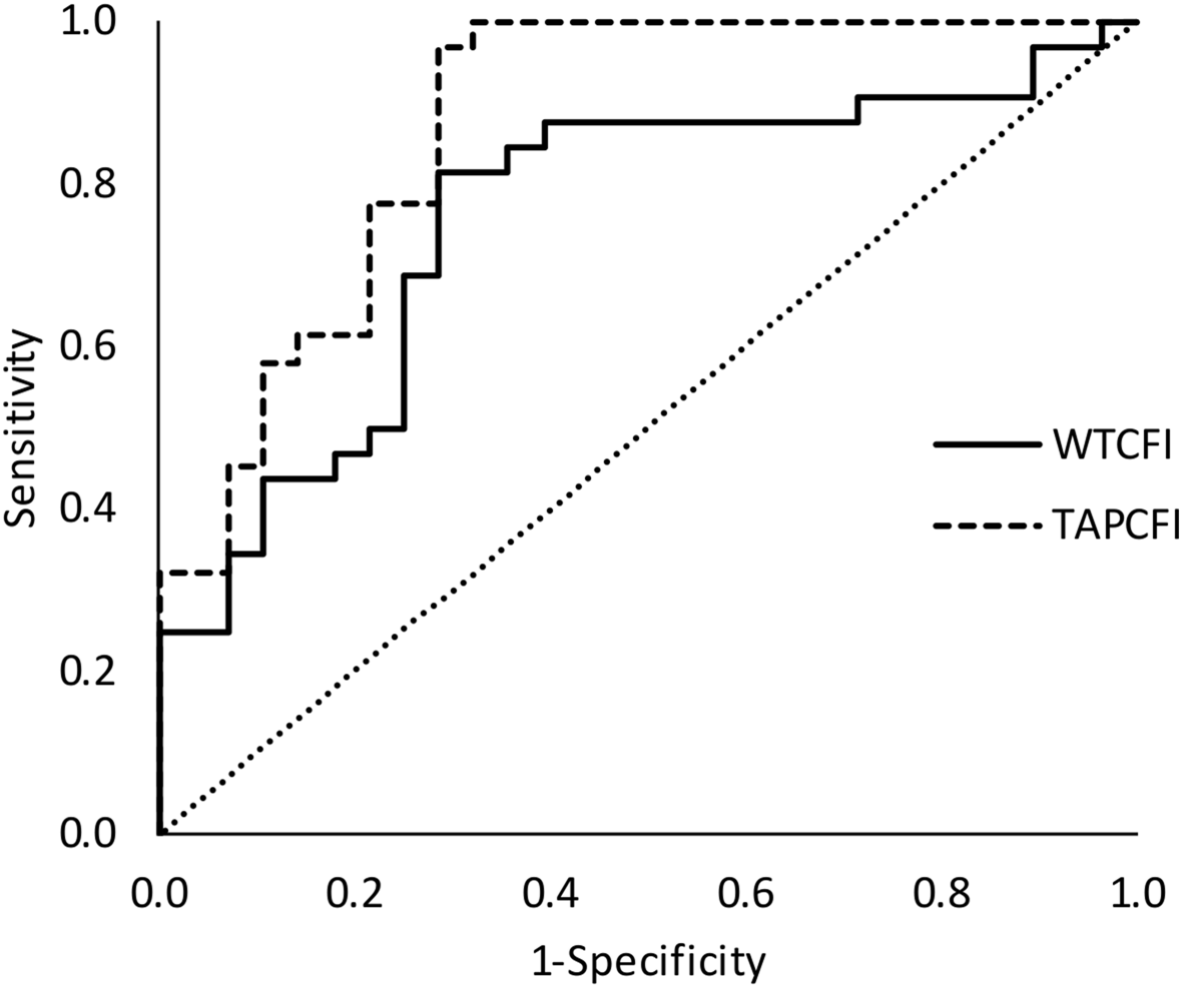
ROC Curves for SDB classification for severe (n: 28) and mild (n: 31) SDB patients according to TAP robustness (TAPCFI, discontinuous line) versus to wrist temperature robustness (WTCFI, continuous line).

The best criteria to discriminate, by means of a decision tree, from mild (n: 31) to severe (n: 28) SDB patients, was TAP robustness (CFI) with a cut-off point of 0.62 yielding an agreement rate of 83.3% (sensitivity = 96.9%; specificity = 67.9%; positive predictive value = 77.5%; negative predictive value = 95.0%). The ROC for TAP robustness (Fig. 4) reached an area under the curve of 0.87 (p < 0.001). Since previously, WT pattern impairment has been reported as changing according to SDB severity^5^, the best WT parameter for SBD classification was also assessed. Thus, WT robustness, as measured by CFI, with a cut-off point of 0.44, yielded an agreement rate of 76.7% (sensitivity = 81.3%; specificity = 71.4%; a positive predictive value = 76.5%; negative predictive value = 76.9%) and the ROC area under the curve was 0.76 (p < 0.01) as it is shown in Fig. 4, that it is, ROC curve for TAP robustness tended to reach better results than for WT robustness (p = 0.067) for discriminating SDB severity.

### Regression analysis for sleep and circadian parameters

Regression analysis was performed to determine the association between sleep and circadian parameters (n: 78). It was controlled for age, sex and BMI. For motor activity (Supplementary Table 1), higher time in N1 stage and CT90 were associated with increased night activity values (L5) and the subsequent decrease of RA. Higher percentage of N3 was correlated with higher RA and lower CT90 was associated to higher CFI. In the case of body position (Supplementary Table 2), MWT-E was positively associated with high IS, RA and CFI whereas a high ODI3 and low NSAT were related with a delay in the nocturnal phase marker (TL5). Finally, the most relevant parameters of TAP variable were significantly associated to decreased nocturnal sleep quality parameters and poorer outcomes of diurnal MWT-E and MSLT-E tests (Supplementary Table 3).

### CPAP treatment

**Figure 5:**
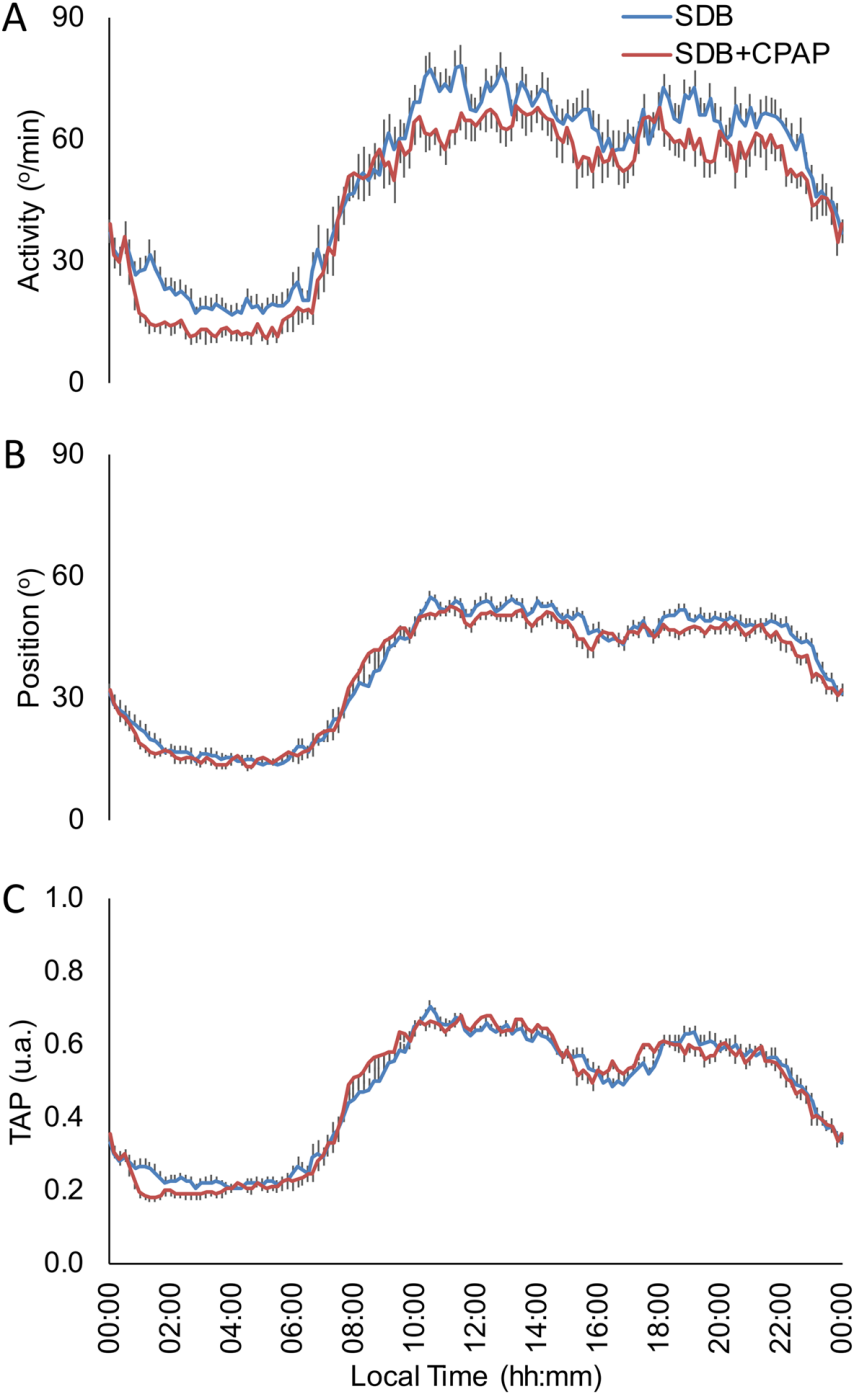
Circadian patterns for 18 SDB subjects at baseline (SDB, red line) and after 6 months of CPAP treatment (SDB + CPAP, in blue). (**A**) Activity (º/min), (**B**) Body Position (º) and (**C**) integrated variable TAP (a. u.). All data are expressed as mean ± SEM.

The influence of CPAP treatment on circadian parameters was addressed by a Mixed Effects Model (n: 18). For the motor activity pattern, CPAP reduced fragmentation (IV), nocturnal activity (L5) and increased day-night contrast (RA) as it is shown in Fig. 5A and Table 3. In addition, diurnal body position (M10) was decreased with the CPAP treatment (Fig. 5B and Table 3). CPAP tended to decrease TAP variable nocturnal values (L5) while increasing day/night contrast (Fig. 5C and Table 3).

**Table 3:** Non-parametrical analysis of CPAP treatment effect on circadian parameters in SDB patients.

Activity		IS	IV	RA	CFI	TL5 (hh:mm)	TM10 (hh:mm)	L5 (^o^/min)	M10 (^o^/min)
	SDB	0.40 ± 0.02	0.78 ± 0.04	0.59 ± 0.03	0.54 ± 0.02	03:58 ± 00:12	15:10 ± 00:27	18.40 ± 1.72	68.92 ± 2.60
	CPAP	0.43 ± 0.02	0.72 ± 0.03	0.68 ± 0.02	0.58 ± 0.02	04:02 ± 00:10	14:45 ± 00:21	12.10 ± 1.30	62.26 ± 3.78
	*p*	*0.139*	*0.016*	*0.011*	*0.061*	*0.794*	*0.455*	*0.003*	*0.112*
Position		IS	IV	RA	CFI	TL5 (hh:mm)	TM10 (hh:mm)	L5 (^o^)	M10 (^o^)
	SDB	0.57 ± 0.03	0.31 ± 0.02	0.55 ± 0.02	0.66 ± 0.02	04:19 ± 00:13	15:02 ± 00:16	14.86 ± 0.98	50.43 ± 1.00
	CPAP	0.54 ± 0.02	0.31 ± 0.01	0.54 ± 0.02	0.64 ± 0.01	04:13 ± 00:12	14:50 ± 00:19	14.51 ± 0.88	48.34 ± 0.97
	*p*	*0.251*	*0.745*	*0.745*	*0.433*	*0.662*	*0.569*	*0.656*	*0.041*
TAP		IS	IV	RA	CFI	TL5 (hh:mm)	TM10 (hh:mm)	L5 (a. u.)	M10 (a. u.)
	SDB	0.55 ± 0.03	0.35 ± 0.02	0.46 ± 0.02	0.61 ± 0.02	03:44 ± 00:11	14:44 ± 00:24	0.23 ± 0.01	0.62 ± 0.01
	CPAP	0.56 ± 0.03	0.33 ± 0.01	0.51 ± 0.02	0.63 ± 0.02	03:16 ± 00:07	14:08 ± 00:16	0.20 ± 0.01	0.62 ± 0.01
	*p*	*0.772*	*0.430*	*0.076*	*0.280*	*0.105*	*1.000*	*0.051*	*0.802*

Interdaily stability (IS); intradaily variability (IV); relative amplitude (RA); circadian function index (CFI); mean of the 10 consecutive hours with the highest values (M10) and its timing (TM10); mean of the 5 consecutive hours with the lowest values (L5) and its timing (TL5). Values are expressed as the mean ± SEM. p denotes significance level (Mixed Model Analysis). In bold are the probability values lower than 0.05.

**Figure 6:**
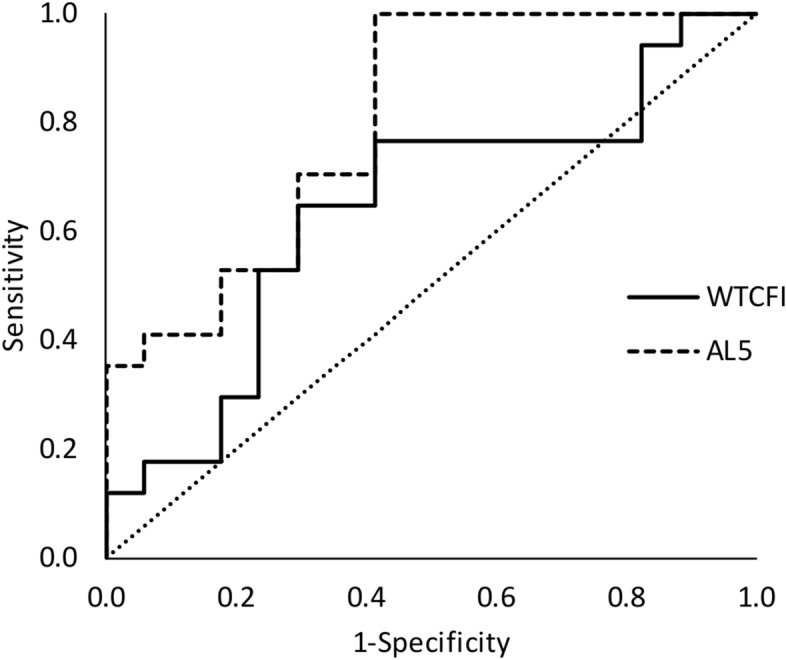
ROC Curves for SDB classification of pre- and after CPAP therapy in severe patients (n: 15) according to their night activity level (AL5, discontinuous line) versus wrist temperature robustness (WTCFI, continuous line).

Finally, the decision tree to discern between pre-treatment and CPAP treated severe patients (n: 15) selected a value of 10.4°/min for nocturnal activity (L5) with an agreement rate of 86.7% (sensitivity = 100.0%; specificity = 73.3%; positive predictive value = 78.9%; negative predictive value = 100.0%). The Fig. 6 shows its corresponding ROC curve reaching an area under the curve of 0.87 (p < 0.01). Again, when comparing with the best WT parameter (CFI), agreement rate was 68.6% with a cut-off point of 0.44 (sensitivity = 66.7%; specificity = 64.7%; positive predictive value = 66.7%; negative predictive value = 64.7%) and the ROC area under the curve was 0.68 (p = 0.11). Then, ROC curve for nocturnal activity (L5) tended to reach better results than for WT robustness (p = 0.063).

## Discussion

Our results highlight that SDB impairs rest-activity, body position and TAP variable circadian rhythms in accordance to SDB severity, whereas CPAP treatment improves motor activity pattern. Besides circadian parameters allow quantitative classification of populations providing a useful clinical screening tool for SDB and CPAP efficacy.

SDB patients showed lower stability and robustness for motor activity and the integrated variable TAP than healthy subjects, conditions related to other sleep disorders^20^, ageing^22^ and cancer chemotherapy circadian impairment^23^. In the case of body position, SDB patients experienced a nocturnal phase advance, a characteristic associated with an aged circadian system^22, 24, 25^, and a more standing position during daytime, probably due to the less pronounced nap period in our sample.

The above-mentioned differences between healthy and SDB subjects´ circadian rhythms can be used as a screening tool for SBD prone population. In a previous paper we showed that SDB patients exhibited lower wrist temperature stability values (IS) with respect to healthy subjects with an agreement rate of 89%, probably due to apnoea-dependent sympathetic activation at night^26^ and the subsequent excessive daytime sleepiness^5^ which can contribute to frequent and unexpected or arrhythmic WT fluctuations.

The severity of SDB, as measured by the Apnea–Hypopnea Index^27^, seems to impair circadian indexes (stability, fragmentation, amplitude and robustness), reinforcing the hypothesis of circadian disruption caused by sleep apnoea^5^, as also occurs with other pathological conditions such as obesity, metabolic syndrome, diabetes, cardiovascular disease or, even, mortality risk^28–31^. Nocturnal values were increased for activity, body position and TAP variable as SDB severity progressed, which indicates a less deep sleep^5, 20, 32^, confirmed by the increase in N1 duration and the decrease of N3 and REM duration. Besides, and according to regression analyses, TAP stability, fragmentation, amplitude, robustness and nocturnal values impairment were related to higher daytime sleepiness as measured by MWT and MSLT as it was previously suggested for WT^5^, and could be a reflect of the sleep structure impairment^15^. Thus, these circadian alterations can be used as a screening procedure to detect individuals prone to suffer SDB before their definitive diagnosis in a sleep unit. Moreover, low TAP robustness, assessed by CFI, allowed distinction in SBD severity reaching an agreement rate of 83%. Given that the CFI variable can be used as an indicator of circadian system status^15^, SDB worsening could implicate a progressive deterioration of the circadian system.

The restorative effect of CPAP treatment has been thoroughly demonstrated for sleepiness^5^ and sleep itself^33, 34^, but also for other alterations such as core body temperature rhythm^6^, cognitive impairment^4^, metabolic syndrome^35^, fibrinolytic^10^ and inflammatory markers^13^, coagulation factors^11^, blood pressure^12^ or autonomic function^36^. Although the main limitation of this study is the absence of the individual adherence and compliance CPAP data, all monitored patients accomplished at least five hours per night during six consecutive weeks^37^ of CPAP treatment, an threshold value for an objective CPAP use that has demonstrated its efficacy for improving nocturnal sleep^34^. In this sense, the activity pattern a great improvement occurred, probably because CPAP treatment reduces nocturnal awakenings^38^ and, hence, would reduce nocturnal activity. Previous studies reported no effect of CPAP treatment^39, 40^, probably due to differences in treatment compliance or monitoring device sensitivity. Activity nocturnal values diminution, and the subsequent increase in amplitude, were compatible with a less fragmented^41^ and deeper sleep due to the reduced number of arousals^5^, as also supported by lesser time in N1 and the AI and SDB indexes improvement. In accordance with the nocturnal decrease in activity level, L5 values can be used to assess CPAP efficacy with an agreement rate of 86%.

In summary, although WT robustness allow us to discern between SDB patients and healthy subjects, when motor activity, body position and TAP robustness are considered reliability for discriminating between mild and severe SDB as well as CPAP usage increases with respect to wrist temperature alone (83% vs 77%, and 87% vs 65%, respectively).

Considering jointly motor activity, body position and WT in the integrated variable TAP reveals that Sleep Disordered Breathing patients present circadian disruption, that increases with the severity of the disease and improves with Continuous Positive Airway Pressure treatment. Thus, TAP variable could constitute, a useful screening tool for SBD prevalence and severity as well as treatment efficacy adherence.

## Materials and methods

In a previous study^5^, we analysed the wrist temperature (WT) circadian pattern in the same group of subjects, using the information from the ACM devices. In the current study we will focus on the information provided by the circadian pattern of motor activity, body position and integrated variable TAP as well as data provided by the Maintenance of Wakefulness Test (MWT), and the Multiple Sleep Latency Test (MSLT).

Ninety-eight consecutive patients with suspected SDB (Table 4) were evaluated at the Hospital Clinic of Barcelona in the Multidisciplinary Sleep Disorders Unit, as previously described in Martinez-Nicolas et al., 2017^5^. The patients were informed about the objectives of the study, the willingness of their participation and that whatever their decision, it would not affect their treatment. Participants who accepted to participate in the study signed an informed consent. Exclusion criteria were being under 18 years of age, use of medications affecting wakefulness or sleep, shift work or irregular sleep–wake schedules during the four weeks before the sleep study, and major medical or psychiatric disorders. Patients with any concomitant sleep disorder other than SDB were excluded by nocturnal polysomnography (PSG).

**Table 4:** Subjects characteristics.

	**Healthy *vs*. SDB**		**SDB severity**			**CPAP Effect**	
	**Healthy**	**SDB**	**Mild**	**Moderate**	**Severe**	**PRE-CPAP**	**CPAP**
Age (y)	51.4 ± 3.2	53.3 ± 1.2	51.2 ± 2.2	56.4 ± 2.1	53.7 ± 1.5	55.3 ± 2.3	55.9 ± 2.3^#^
Sex (M/W)	18/14	57/21	18/13	16/3	23/5	16/2	16/2
BMI (kg/m^2^)	24.7 ± 0.7	30.0 ± 0.6*	27.3 ± 0.6	29.7 ± 1.2	33.4 ± 1.1^ab^	31.3 ± 1.2	31.6 ± 1.1

Healthy: control group, n: 32 SDB: Sleep Disordered Breathing, n: 78; Snorer/Mild: subjects with less than 15 events/hour (n: 31); Moderate: between 15 and 30 events/hour (n: 19); Severe: more than 30 events/hour (n: 28). Pre-CPAP and CPAP: SDB subgroup before and after CPAP treatment (n: 18). BMI: Body Mass Index. All data are expressed as mean ± SEM, except for the men/women ratio. *Indicates statistical differences between healthy and SDB patients (General Linear Model, p < 0.05). For mild, moderate and severe SDB groups, “a” indicates statistical differences when compared to mild SDB patients, “b” indicates statistical differences with moderate SDB patients (General Linear Model, p < 0.05, Bonferroni’s post hoc). # Denotes statistical differences between pre-CPAP and post-CPAP treatment (Mixed Model Analysis, p < 0.05).

A total of 32 healthy subjects were recruited (age and gender balanced with the SDB patients) at the Chronobiology Lab of the University of Murcia (Table 4). They are included in this study in order to illustrate healthy circadian patterns for activity, body position and TAP. Exclusion criteria were the same as those applied to the SDB patients, but also included snoring (as reported during a personal interview) and/or excessive daytime sleepiness (Epworth Sleepiness Scale score higher than 12)^42^.

The study follows the bioethical principles set out by the Declaration of Helsinki. Data from the volunteers were protected according to Spanish Law 15/1999 from 13 September. The study was approved by the Hospital Clinic of Barcelona and the University of Murcia ethics committees for the SDB patients and healthy subjects, respectively.

### Design

Healthy volunteers were monitored for an entire week whereas SDB patients were monitored 6 days under free-living conditions and an additional day, the last one, under controlled conditions in hospital. During this week, the subjects were encouraged to maintain their habitual life style. Since PSG and nap-protocols could interfere with the normal sleep pattern of the patients^43^, both test were performed at the end of the monitoring period, allowing at least 6 days of continuous recordings (the minimum for a reliable non-parametrical analysis, according to literature^44^).

For ambulatory circadian monitoring, all subjects wore a Thermochron iButton DS1921H (Maxim Integrated Products, Sunnyvale, CA) programmed to sample every 10 min over the whole week to measure WT^32^, placed on the wrist of the non-dominant hand over the radial artery as already described^5, 45–47^, and isolated from the environment by a double-sided cotton sport wrist band^32^. In addition, every subject was monitored with a HOBO Pendant G Acceleration Data Logger UA- 004–64 actimeter (Onset Computer, Bourne, MA) programmed every 30 s, positioned on the non-dominant arm by means of a sport band for motor activity and body position monitoring as already described^15, 20, 45^. Briefly, motor activity was measured as the rate of change in degrees per minute and position represents the angle between the X-axis of the accelerometer (parallel to the humerus bone of the arm) and the horizontal plane. Data from activity and body position were averaged for 10-min intervals to facilitate WT comparisons. Wrist **T**emperature, motor **A**ctivity and body **P**osition were combined in order to obtain the integrated variable TAP, according to the algorithms previously described^15^. In summary, and as previously described^5, 15, 20–22^, each variable was normalized between 0 and 1 and averaged to obtain the integrated variable (TAP). Thus, a TAP value of 1 indicates the lowest values of WT, the highest values of activity and a standing position (compatible with wake periods), whereas a score of 0 corresponds to the highest WT values, the lowest activity values and a horizontal position (compatible with sleep periods)^15^.

On the last day of the week, patients with suspected SDB underwent a 24-h sleep study, including questionnaires and neurophysiological tests as previously described^5^. After admission, the Epworth Sleepiness Scale (ESS) and Barcelona Sleepiness Index (BSI) were used to assess subjective daytime sleepiness^42, 48^. Nocturnal PSG was performed according to standard practice parameters and diagnostic criteria^49, 50^ with sleep stages independently and manually double scored according to the American Academy of Sleep Medicine (AASM) criteria, using 30 s epochs^51^. The nap protocol was initiated the morning after PSG in order to objectively measure daytime sleepiness throughout the day (review Guaita et al., 2015 for further details^48^). In brief, we used the research version protocol comprising 5 sets of the Maintenance of Wakefulness Test (MWT), the Multiple Sleep Latency Test (MSLT) that started at 08:30 and every 2 h onwards^52^. Each nap set was preceded by a measurement of vigilance with Sustained Attention to Response Task (SART), with a duration of 4 min^53^ and using commission errors, missed errors and total errors^54^. Due to their skewed distribution, MWT and MSLT were log transformed previously to statistical analysis.

### Continuous positive airway pressure treatment

From the cohort of 78 patients, eighteen moderate or severe SDB patients with resistant hypertension or excessive daytime sleepiness were treated with CPAP and evaluated again following the same protocol (nocturnal PSG followed by SART-MSLT-MWT protocol). CPAP titration was performed following the recommendations of the Spanish Sleep Society^55^. CPAP compliance was measured objectively using a built-in CPAP meter. For the analysis, five hours per night, measured by the CPAP, during six consecutive weeks were considered the strict minimum use of CPAP^37^.

### Data analysis

The WT data were obtained, filtered and processed from data previously reported^5^. The mean daily patterns for motor activity, body position and TAP variable were calculated per individual, and then averaged per group.

In order to characterize the aforementioned circadian rhythms a nonparametric analysis was performed as previously described^5^. This analysis determines the following parameters: interdaily stability (the constancy of the 24-h rhythmic pattern over days, IS), intradaily variability (rhythm fragmentation, IV), relative amplitude (RA) and circadian function index (CFI) calculated by the integration of IS, IV and RA oscillating between 0 (absence of circadian rhythmicity) and 1 (robust circadian rhythm)^15, 56^. RA was calculated as the difference between M10 (average of 10-min intervals for the 10 consecutive hours with maximum values) and L5 (average of 10-min intervals for the 5 consecutive hours of minimum values) divided by the sum of M10 and L5 for variables with acrophase during daytime (activity, body position and TAP), as previously published^41, 56^. The timing for L5 and M10 were used as nocturnal and diurnal phase markers (TL5 and TM10, respectively).

Circadian variables parameters in healthy subjects and SDB patients were compared using a General Linear Model controlled by age, gender and body mass index (BMI). Differences between snorers/mild SDB (AHI < 15 events/hours), moderate SDB (AHI between 15 and 30 events/hour) and severe SDB (AHI > 30 events/hour) were assessed by a General Linear Model controlled by age, gender and BMI (followed by post hoc pairwise comparisons and a Bonferroni test). In addition, a regression analysis was performed between sleep and circadian parameters controlling again for age, gender and BMI (with Bonferroni correction for multiple comparisons). A Mixed Effects Model was performed to determine the effect of CPAP treatment on SDB patients.

Circadian parameters for WT (showed in a previous work^5^), activity, body position and TAP were entered in WEKA version 3.8.3 (University of Waikato, Hamilton, New Zealand)^57^. An independent classification was then performed to discern between i) healthy subjects and SDB patients, ii) mild and severe SDB patients and iii) pre-treatment and CPAP therapy groups by means of a J.4.8 decision tree that uses the C 4.5 algorithm for decision making^58^, which selects the decision that maximizes information gain at each step. In order to simplify the algorithm, all decision trees were required to use only one decision. Each individual decision tree corresponds to the best of 100 iterations performed with 66% of the data randomly selected and checked against the other 33%. Then, specificity (test’s ability to detect correctly (i) SDB patients, (ii) severe SDB patients and (iii) pre-treatment group), sensitivity (test’s ability to detect correctly (i) healthy subjects, (ii) mild SDB patients and (iii) CPAP therapy group), positive predictive value (proportion of positive results that are true positive; the positive results were (i) SDB, (ii) severe SDB patients and (iii) pre-treatment patients), negative predictive value (proportion of negative results that are true negative; the negative results were (i) healthy subjects, (ii) mild SDB patients and (iii) CPAP therapy group), agreement rate and ROC curve for every decision rule were calculated, which were compared by a Wilcoxon test. Data were processed using Microsoft Office Excel 2016, and all statistical analyses were performed using SPSS version 23.0 software (SPSS, Chicago, Illinois, USA). Values of p < 0.05 were considered to be statistically significant for General Linear Model and Wilcoxon test and p < 0.00625 for Regression analysis.

## Data availability statement

The raw data supporting the conclusions of this manuscript are available on request to the corresponding author.

## Supplementary Information

Supplementary Information.
