# Supplementary material for: Ambulatory circadian monitoring in sleep disordered breathing patients and CPAP treatment
Source: Sci Rep. 2021 Jul 19;11:14711. doi: 10.1038/s41598-021-94315-0 (PMC8290024; doi:10.1038/s41598-021-94315-0)
Supplement: Supplementary file 1 — Supplementary Information. [file 41598_2021_94315_MOESM1_ESM.docx]

| PSG-SART-MWT-MSLT\ACT | | IS | IV | RA | CFI | M10 | L5 | TM10 | TL5 |
| --- | --- | --- | --- | --- | --- | --- | --- | --- | --- |
| N1 (%) |  |  |  | **-0.332** |  |  | **0.386*** |  |  |
| N2 (%) |  |  |  |  |  |  |  |  |  |
| N3 (%) |  | 0.266 |  | **0.331** | 0.285 |  | -0.290 |  |  |
| REM (%) |  |  |  |  |  |  |  |  |  |
| Rem Episodes |  |  |  |  |  |  |  |  |  |
| AI (events/h) |  |  |  |  |  |  |  |  |  |
| AHI (events/h) |  |  |  |  |  |  |  |  |  |
| CT90 (min) |  | -0.253 | 0.253 | **-0.375*** | **-0.335*** |  | **0.330*** |  |  |
| ODI3 (events/h) |  |  |  |  |  |  | 0.269 |  |  |
| MSAT (%) |  |  |  |  |  |  |  |  |  |
| NSAT (%) |  |  |  | 0.206 |  |  | -0.253 |  | -0.238 |
| ESS (a.u.) |  |  |  |  |  |  |  |  |  |
| BSI (a.u.) |  |  |  |  |  |  |  |  |  |
| MWT-SL (min) |  |  |  |  |  |  |  |  | -0.237 |
| MWT-E (min) |  |  |  |  |  |  |  |  |  |
| MSLT-SL (min) |  |  |  | 0.287 |  |  | -0.311 |  |  |
| MSLT-E (min) |  |  |  | 0.240 |  |  | -0.252 |  |  |
| SART-CE (counts) |  |  |  |  |  |  |  |  |  |
| SART-ME (counts) |  |  |  |  |  |  |  |  |  |
| SART-CE (counts) |  |  |  |  |  |  |  |  |  |

**Supplementary Table 1.** Lineal regression analysis between **activity** circadian parameters and sleep parameters (n: 78). Interdaily stability (IS); intradaily variability (IV); relative amplitude (RA); circadian function index (CFI); mean of the 5 consecutive hours with the highest values (M5) and its timing (TM5); mean of the 10 consecutive hours with the lowest values (L10) and its timing (TL10), N1: Sleep NREM phase 1; N2: Sleep NREM phase 2; N3: Sleep NREM phase 3; REM: Sleep REM phase; REM Episodes: number of REM Episodes; AI: Arousal Index; AHI: Apnoea/Hypopnoea Index; CT90: cumulative time spent with oxygen saturation lower than 90%; ODI3: Oxygen Desaturation Index 3%; MSAT: Mean saturation of oxygen expressed in percentage; NSAT: Nadir of oxygen saturation expressed in percentage; ESS: Epworth Sleepiness Scale; BSI: Barcelona Sleepiness Scale; a. u.: arbitrary units; MWT-SL: Maintenance of Wakefulness Test-Sustained Sleep Latency; MWT-E: Maintenance of Wakefulness Test-1st Epoch Sleep Latency; MSLT-SL: Multiple Sleep Latency Test-Sustained Sleep Latency; MSLT-E: Multiple Sleep Latency Test-1st Epoch Sleep Latency; SART-CE: Sustained Attention to Response Task-Commission Errors; SART-ME: Sustained Attention to Response Task-Missed Errors; SART-TE: Sustained Attention to Response Task-Total Errors. Only significant regressions are shown; however, in bold are the probability values lower than 0.00625, *indicates a significance level <0.00125 (Linear regression controlled for gender, age and BMI).

| PSG-SART-MWT-MSLT\POS | | IS | IV | RA | CFI | M10 | L5 | TM10 | TL5 |
| --- | --- | --- | --- | --- | --- | --- | --- | --- | --- |
| N1 (%) |  |  |  |  |  |  |  | -0.300 |  |
| N2 (%) |  |  |  |  |  |  |  |  |  |
| N3 (%) |  |  |  |  |  |  |  |  | -0.261 |
| REM (%) |  |  |  | 0.222 |  |  | -0.255 |  | -0.236 |
| Rem Episodes |  |  |  |  |  |  |  |  |  |
| AI (events/h) |  |  |  |  |  |  | 0.235 |  | 0.229 |
| AHI (events/h) |  |  |  |  |  |  | 0.229 |  | 0.263 |
| CT90 (min) |  |  |  |  |  |  |  |  |  |
| ODI3 (events/h) |  |  |  |  |  |  | 0.250 |  | **0.309** |
| MSAT (%) |  |  |  |  |  |  |  |  |  |
| NSAT (%) |  |  |  |  |  | -0.227 |  |  | **-0.334** |
| ESS (a.u.) |  |  |  |  |  |  |  |  |  |
| BSI (a.u.) |  |  |  |  |  |  |  |  |  |
| MWT-SL (min) |  | 0.285 |  | 0.281 | 0.275 |  |  |  |  |
| MWT-E (min) |  | **0.398*** |  | **0.403*** | **0.403*** |  | -0.268 |  |  |
| MSLT-SL (min) |  | 0.275 |  |  | 0.237 |  |  |  |  |
| MSLT-E (min) |  | 0.245 |  | 0.240 | 0.246 |  | -0.252 |  |  |
| SART-CE (counts) |  |  |  |  |  |  |  |  |  |
| SART-ME (counts) |  |  |  |  |  |  |  |  |  |
| SART-CE (counts) |  |  |  |  |  |  |  |  |  |

**Supplementary Table 2.** Lineal regression analysis between **body** **position** circadian parameters and sleep parameters (n: 78). Interdaily stability (IS); intradaily variability (IV); relative amplitude (RA); circadian function index (CFI); mean of the 5 consecutive hours with the highest values (M5) and its timing (TM5); mean of the 10 consecutive hours with the lowest values (L10) and its timing (TL10), N1: Sleep NREM phase 1; N2: Sleep NREM phase 2; N3: Sleep NREM phase 3; REM: Sleep REM phase; REM Episodes: number of REM Episodes; AI: Arousal Index; AHI: Apnoea/Hypopnoea Index; CT90: cumulative time spent with oxygen saturation lower than 90%; ODI3: Oxygen Desaturation Index 3%; MSAT: Mean saturation of oxygen expressed in percentage; NSAT: Nadir of oxygen saturation expressed in percentage; ESS: Epworth Sleepiness Scale; BSI: Barcelona Sleepiness Scale; a. u.: arbitrary units; MWT-SL: Maintenance of Wakefulness Test-Sustained Sleep Latency; MWT-E: Maintenance of Wakefulness Test-1st Epoch Sleep Latency; MSLT-SL: Multiple Sleep Latency Test-Sustained Sleep Latency; MSLT-E: Multiple Sleep Latency Test-1st Epoch Sleep Latency; SART-CE: Sustained Attention to Response Task-Commission Errors; SART-ME: Sustained Attention to Response Task-Missed Errors; SART-TE: Sustained Attention to Response Task-Total Errors. Only significant regressions are shown; however, in bold are the probability values lower than 0.00625, *indicates a significance level <0.00125 (Linear regression controlled for gender, age and BMI).

| PSG-SART-MWT-MSLT\TAP | | IS | IV | RA | CFI | M10 | L5 | TM10 | TL5 |
| --- | --- | --- | --- | --- | --- | --- | --- | --- | --- |
| N1 (%) |  |  |  |  |  |  | 0.228 |  |  |
| N2 (%) |  |  |  |  |  |  |  |  |  |
| N3 (%) |  |  |  |  |  |  |  |  |  |
| REM (%) |  |  |  |  |  |  |  |  |  |
| Rem Episodes |  |  |  |  |  |  |  |  |  |
| AI (events/h) |  |  | **0.338** | **-0.397*** | **-0.378*** |  | **0.404*** |  |  |
| AHI (events/h) |  | **-0.402*** | 0.232 | **-0.450#** | **-0.456#** |  | **0.432#** |  |  |
| CT90 (min) |  |  | 0.245 |  |  |  |  |  |  |
| ODI3 (events/h) |  |  | **0.317** | **-0.308** | **-0.330** |  | **0.314** |  | 0.238 |
| MSAT (%) |  |  |  |  |  |  |  |  |  |
| NSAT (%) |  |  |  |  |  |  | -0.224 |  |  |
| ESS (a.u.) |  |  |  |  |  |  |  |  |  |
| BSI (a.u.) |  |  |  |  |  |  |  |  |  |
| MWT-SL (min) |  | 0.278 | -0.280 | 0.276 | 0.298 |  | -0.277 |  | -0.344 |
| MWT-E (min) |  | **0.400*** | -0.309 | **0.304** | **0.336*** |  | **-0.309** |  | **-0.335** |
| MSLT-SL (min) |  | 0.279 | **-0.316** | **0.326** | **0.329** |  | **-0.332** |  |  |
| MSLT-E (min) |  | 0.247 | -0.253 | 0.262 | 0.274 |  | -0.273 | -0.241 |  |
| SART-CE (counts) |  |  |  |  |  |  |  |  |  |
| SART-ME (counts) |  |  |  |  |  |  |  |  |  |
| SART-CE (counts) |  |  |  |  |  |  |  |  |  |

**Supplementary Table 3.** Lineal regression analysis between **TAP** variable circadian parameters and sleep parameters (n: 78). Interdaily stability (IS); intradaily variability (IV); relative amplitude (RA); circadian function index (CFI); mean of the 5 consecutive hours with the highest values (M5) and its timing (TM5); mean of the 10 consecutive hours with the lowest values (L10) and its timing (TL10), N1: Sleep NREM phase 1; N2: Sleep NREM phase 2; N3: Sleep NREM phase 3; REM: Sleep REM phase; REM Episodes: number of REM Episodes; AI: Arousal Index; AHI: Apnoea/Hypopnoea Index; CT90: cumulative time spent with oxygen saturation lower than 90%; ODI3: Oxygen Desaturation Index 3%; MSAT: Mean saturation of oxygen expressed in percentage; NSAT: Nadir of oxygen saturation expressed in percentage; ESS: Epworth Sleepiness Scale; BSI: Barcelona Sleepiness Scale; a. u.: arbitrary units; MWT-SL: Maintenance of Wakefulness Test-Sustained Sleep Latency; MWT-E: Maintenance of Wakefulness Test-1st Epoch Sleep Latency; MSLT-SL: Multiple Sleep Latency Test-Sustained Sleep Latency; MSLT-E: Multiple Sleep Latency Test-1st Epoch Sleep Latency; SART-CE: Sustained Attention to Response Task-Commission Errors; SART-ME: Sustained Attention to Response Task-Missed Errors; SART-TE: Sustained Attention to Response Task-Total Errors. Only significant regressions are shown; however, in bold are the probability values lower than 0.00625, *indicates a significance level <0.00125 (Linear regression controlled for gender, age and BMI).
